# Long-term outcomes of oral immunotherapy for anaphylactic egg allergy in children

**DOI:** 10.1016/j.jacig.2022.03.005

**Published:** 2022-04-30

**Authors:** Koki Sasamoto, Noriyuki Yanagida, Ken-ichi Nagakura, Makoto Nishino, Sakura Sato, Motohiro Ebisawa

**Affiliations:** a Department of Pediatrics, National Hospital Organization, Sagamihara National Hospital, Kanagawa, Japan; c Clinical Research Center for Allergy and Rheumatology, National Hospital Organization, Sagamihara National Hospital, Kanagawa, Japan; b Department of Pediatrics, Toho University Ohashi Medical Center, Tokyo, Japan

**Keywords:** Anaphylaxis, desensitization, egg allergy, food allergy, IgE, long-term, oral food challenge, oral immunotherapy

## Abstract

**Background:**

Studies of long-term oral immunotherapy (OIT) in children with anaphylactic egg allergy are limited.

**Objective:**

Our aim was to investigate the long-term outcomes of OIT for anaphylactic egg allergy.

**Methods:**

The participants included children (aged ≥ 5 years) with a history of anaphylaxis in response to eggs and objective reactions to oral food challenge (OFC) with 250 mg of egg protein. In the OIT group, the home starting dose of egg protein set during 5 days of hospitalization was ingested once daily and gradually increased to 1000 mg. Over the next year, participants temporarily discontinued OIT for 2 weeks and underwent OFC with 3100 mg of egg protein annually until they passed. The historical control group comprised patients who did not receive OIT and repeated OFCs annually.

**Results:**

In the OIT group (n = 20), the baseline median egg white– and ovomucoid-specific IgE levels were 45.5 and 38.5 kU_A_/L, respectively. The rate of passing OFC with 3100 mg of egg protein gradually increased in the OIT group, with rates of 20% at 1 year, 35% at 2 years, and 55% at 3 years, which were significantly higher than the rates in the historical control group at 3 years (5% [*P* < .001]). In the OIT group, 5 anaphylaxis events (0.04%) occurred at home, and 1 participant required intramuscular adrenaline. Furthermore, egg white- and ovomucoid-specific IgE levels decreased significantly after 3 years in both groups, whereas in the OIT group, these specific IgG and IgG_4_ levels increased significantly after a year.

**Conclusion:**

Long-term OIT accelerated immunologic changes and enabled ingestion of 3100 mg of egg protein in half of the participants with anaphylactic egg allergy.

## Graphical abstract

**Figure:**
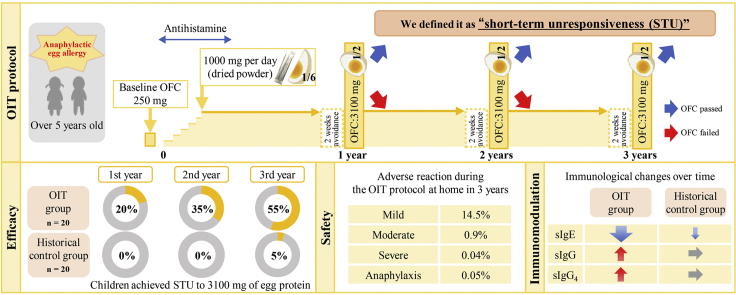

Hen’s egg allergy is one of the most common food allergies in children.^1^^,^^2^ A recent review of egg allergy reported that eggs triggered allergies in 7% to 12% of patients with pediatric anaphylaxis.^3^ Moreover, a cross-sectional survey in the United States reported that more than 25% of children with egg allergies had experienced severe allergic reactions to egg exposure.^4^ The majority of children with egg allergy acquire tolerance as they age^5^; however, children with severe egg allergy, such as those with high levels of egg white–specific IgE or a history of egg anaphylaxis, have difficulty in acquiring tolerance.^6^^,^^7^ Because eggs are frequently included in processed foods and are difficult to avoid, the risk of accidental exposure is high. Therefore, children who have severe egg allergy experience a significantly decreased quality of life.^4^ Recently, oral immunotherapy (OIT) for food allergies has effectively increased the reaction threshold to allergenic foods.^8^, ^9^, ^10^, ^11^, ^12^, ^13^, ^14^, ^15^, ^16^, ^17^, ^18^, ^19^, ^20^, ^21^, ^22^, ^23^, ^24^, ^25^, ^26^, ^27^, ^28^ However, egg OIT has been studied mostly in the short term (ie, < 1 year).^15^, ^16^, ^17^, ^18^, ^19^, ^20^, ^21^, ^22^ The only 2 reports on long-term egg OIT reported study periods as 4 and 2 years, respectively.^16^^,^^21^ They excluded participants who developed severe anaphylaxis, and they did not describe the proportion of participants with a history of anaphylaxis. Thus, in our study, we clarified the long-term outcome of OIT in children with anaphylactic egg allergy for the first time.

## Methods

### Study design and participants

This single-center study was a prospective, nonrandomized controlled trial from conducted from January 2014 to June 2016 at Sagamihara National Hospital (University Hospital Medical Information Network Registry identifier UMIN000011202) and was conducted in accordance with the principles of the Declaration of Helsinki, with approval from the ethical committee of National Sagamihara Hospital (approval no. 2013070916).

**Fig 1:**
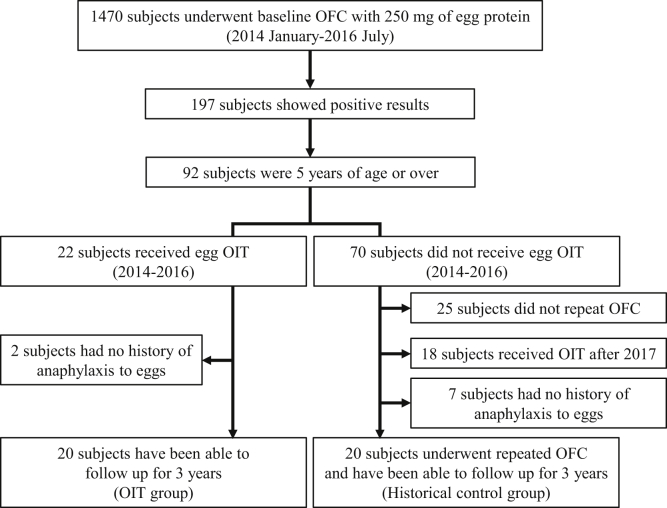
Study design.

The participants were children aged 5 years or older who had a history of anaphylaxis in response to eggs and were positive for oral food challenge (OFC) with 250 mg of egg protein (Fig 1). The exclusion criteria included poorly controlled bronchial asthma, atopic dermatitis, or participation in immunotherapy for other antigens. Among the participants who satisfied the eligibility criteria, those who received OIT constituted the OIT group, whereas the historical control group included the remaining patients who did not desire OIT and received OFC yearly for up to 3 years. We obtained informed consent from the guardians of all study participants.

### OFC protocol

For the baseline OFC, we used cooked egg powder containing 250 mg of egg protein.^29^^,^^30^ We performed OFC by administering 2 doses 1 hour apart. The initial dose was one-quarter of 250 mg of egg protein, and the second was three-quarters of the total dose.

OFC with 3100 mg of egg protein (equivalent to half an egg) to evaluate short-term unresponsiveness (STU) after 2 weeks of egg avoidance was also performed by using cooked egg powder, pumpkin cake, or hamburger steak containing 3100 mg of egg protein; basically, OFC was performed with cooked egg powder. Cake and hamburger were used only if the participants found the taste of the powder to be unacceptable. These items were provided in quarters, quarters, and halves, respectively, every 60 minutes. An OFC response was defined as positive when objective symptoms occurred. The severity of symptoms was assessed as mild, moderate, or severe according to the Japanese Food Allergy Guidelines (see Table E1 in the Online Repository at www.jaci-global.org).^31^ Anaphylaxis was defined according to the World Allergy Organization guidelines.^32^^,^^33^

### OIT protocol

**Fig 2:**
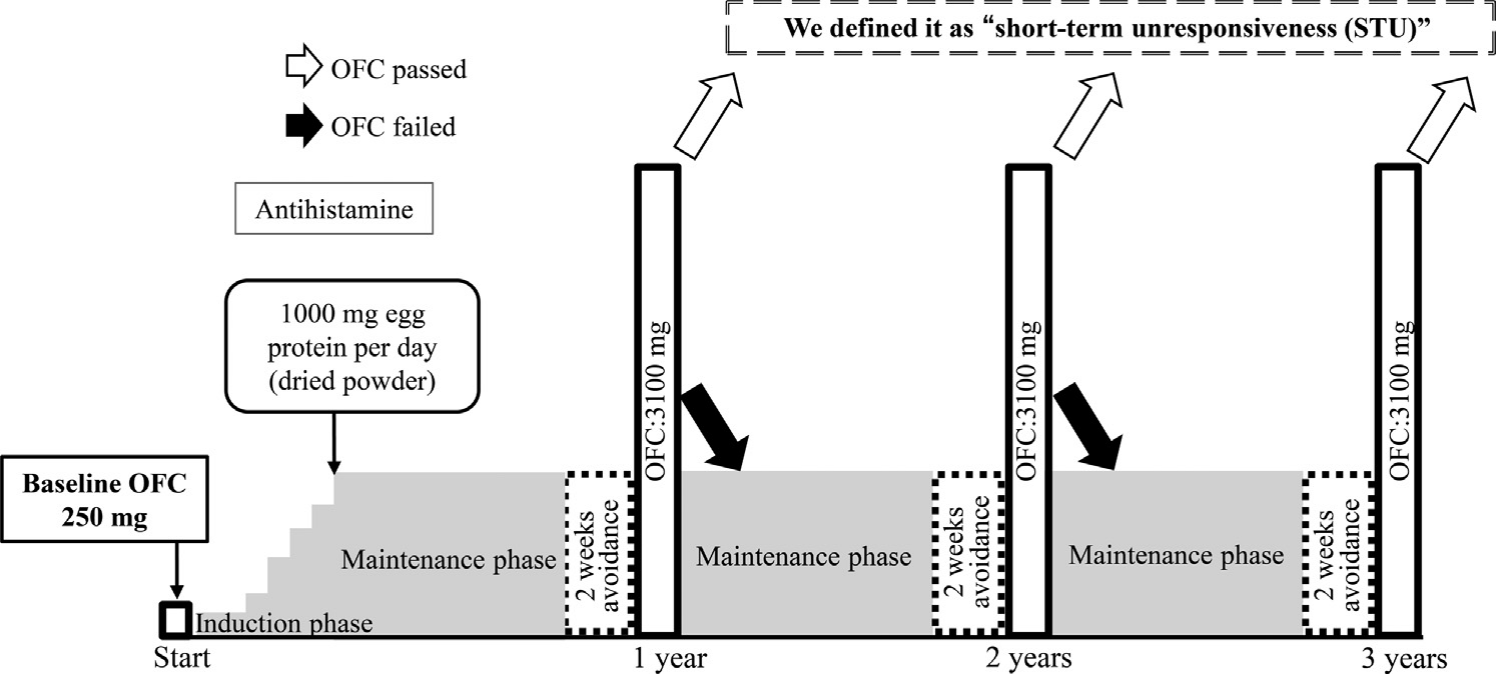
OIT protocol.

The OIT protocol is illustrated in Fig 2. The participants were premedicated with an antihistamine during a 5-day admission, and the home starting dose of egg protein was determined according to an 8-step dosing schedule (62.5-1000 mg) (see Table E2 in the Online Repository at www.jaci-global.org). During hospitalization, participants consumed cooked egg powder daily; if there were mild or no symptoms, the participants ingested the same dose the next day. If the participants developed moderate or severe adverse reactions, their dose was reduced by 1 or 2 steps, respectively, and they were discharged after having been confirmed to be asymptomatic. After discharge, the home starting dose was continued once a day for 1 month while the participant was taking an antihistamine. One month later, if a participant could ingest cooked egg powder without developing any symptoms for 5 consecutive days, the home ingestion dose was increased by 1 step, up to 1000 mg (the maintenance dose). As was the case during hospitalization, the dose was reduced if the participants developed adverse reactions at home. Premedication with an antihistamine was terminated when a participant remained asymptomatic for 1 month with daily ingestion of 1000 mg of egg protein. After 1 year of OIT, if a participant was asymptomatic after taking 1000 mg of egg protein for more than 3 months, they underwent OFC with 3100 mg of egg protein during hospitalization after 2 weeks of complete egg avoidance. Participants who passed OFC with 3100 mg of egg protein were permitted to ingest processed foods containing less than 3100 mg of egg protein twice a week at home. If a participant failed OFC, they resumed daily consumption of 1000 mg of egg protein with cooked egg powder the next day and underwent OFC annually.

All participants were prescribed antihistamines, steroids, inhaled β_2_ stimulants, and autoinjectable adrenaline for the treatment of adverse reactions. During hospitalization, the children and parents were instructed to use or administer the aforementioned treatments as needed in accordance with the Japanese Guidelines for Food Allergy.^14^^,^^31^^,^^34^ They were instructed to record the details of their daily intake and symptoms in a diary. To manage the adverse reaction of eosinophilic esophagitis, we checked in the outpatient clinic for the appearance of persistent abdominal pain, nausea, vomiting, and anorexia. Furthermore, we provided direct support by telephone 24 hours a day. At least once every 3 months, the attending physician checked the participants for adverse reactions, ingestion doses, and adherence to the protocol in the outpatient department.

### Historical control group

**Fig E1:**
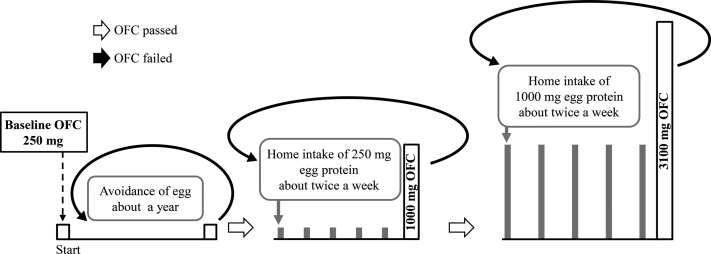


A flowchart of the historical control group is shown in Fig E1 (see the Online Repository at www.jaci-global.org). Those patients in the historical control group who had not participated in other studies were instructed to eliminate eggs for 1 year, after which an OFC of 250 mg of egg protein was performed. If they passed the 250-mg OFC at 1 year, they ingested processed food containing 250 mg of egg protein at home and underwent an OFC with 1000 mg of egg protein after 3 to 6 months. If they did not pass OFC with 250 mg of egg protein, they were instructed to completely eliminate eggs again and were reevaluated with a 250-mg OFC after 1 year. When a patient passed the 1000-mg OFC, an OFC with 3100 mg of egg protein was performed after 3 to 6 months. This way, a stepwise OFC was performed,^31^ and the threshold for eggs after 3 years was confirmed.

### Cooked egg powder

The powder was manufactured by Kewpie Corporation (Tokyo, Japan) and contained 250 mg of egg protein in 1 packet (1 g), which is equivalent to 1/25th of a whole egg. The powder was produced by boiling eggs at 95°C for 15 minutes, pasteurization at 65°C for 20 minutes, and subsequent spray drying. The powder was dissolved in apple juice. When used in OFC, this powder showed the same antigen levels as the egg-containing cake, as previously reported.^29^^,^^30^

### Evaluation of immunologic markers

We measured both egg white- and ovomucoid-specific IgE levels at baseline and every 12 months in the OIT group and the historical control group. Egg white- and ovomucoid-specific IgG and IgG_4_ (ImmunoCAP assay system, Thermo Fisher Scientific, Uppsala, Sweden) levels were measured at baseline and 12 months in both groups.

### Definition of terms

Desensitization was defined as the absence of symptoms after ingestion of 1000 mg of egg protein on consecutive days. After 1 year of OIT, passing OFC with 3100 mg of egg protein after 2 weeks of avoidance of eggs was deemed achievement of STU. If a participant achieved STU to 3100 mg of egg protein, we permitted them to consume processed foods with up to 3100 mg of egg protein at home more than twice a week, which allowed consumption of many processed foods. We considered the participants' risk of symptoms due to processed foods to have been lowered^35^ and believed that their quality of life could be further improved.^4^

### Outcomes

The primary outcome of this study was the proportion of participants who passed OFC with 3100 mg of egg protein after 2-week avoidance within 3 years. The secondary outcomes were changes in immunologic markers and adverse reactions.

### Statistical analysis

The results of the analyses are presented as medians and ranges. Differences between groups were analyzed by using the Fisher exact test and the Mann-Whitney *U* test. The Wilcoxon signed rank test was used to examine changes in the serum levels of antibodies. All analyses were performed with a 2-tailed test, and *P* values less than .05 were considered statistically significant. Statistical analyses were performed with SPSS, version 24 (IBM Corp, Armonk, NY).

## Results

### Patient background

Of the 92 patients who were positive for OFC with 250 mg of egg protein, 20 who had a history of anaphylaxis in response to eggs and desired OIT were enrolled in the OIT group. Regarding the historical control group, we excluded 18 patients who started OIT after 2017, 25 patients in whom the egg threshold after 3 years could not be evaluated, and 9 patients with no history of anaphylaxis in response to eggs. Thus, we ultimately included 20 patients in the historical control group (Fig 1). The median age at baseline was significantly higher in the OIT group (8.0 years) than in the historical control group (6.0 years). In the OIT group, the median baseline OFC threshold was 140 mg of egg protein, and the median egg white- and ovomucoid-specific IgE levels were 45.5 and 38.5 kU_A_/L, respectively. There were no significant differences between the groups in any terms of any characteristic other than age (Table I).

**Table I:** Baseline characteristics

Characteristic	OIT group (n = 20)	Historical control group (n = 20)	*P* value
Age (y), median (IQR)	8.0 (6-10)	6.0 (5-8)	.03
Sex (male), no. (%)	13 (65%)	13 (65%)	>.99
Complications, no. (%)			
Bronchial asthma	15 (75%)	12 (60%)	.50
Atopic dermatitis	14 (70%)	13 (65%)	>.99
Allergic rhinitis	9 (45%)	10 (50%)	>.99
Baseline OFC			
Threshold dose (mg), median IQR	140 (2-250)	192 (2-250)	>.99
Severity, no.			.56
Mild	3	5	
Moderate	16	13	
Severe	1	2	
Total IgE level (IU/mL), median IQR	751 (492-1432)	903 (532-2272)	.47
Specific IgE level (kU_A_/L), median IQR			
Egg white	45.5 (12.7-94.7)	46.5 (15.0-66.3)	.57
Ovomucoid	38.5 (13.5-75.5)	26.0 (16.2-62.1)	.53
Specific IgG level (mg_A_/L), median IQR			
Egg white	6.7 (3.8-15.3)	3.6 (3.1-6.4)	.09
Ovomucoid	6.9 (3.3-9.5)	3.3 (3.0-5.8)	.16
Specific IgG_4_ level (mg_A_/L), median IQR			
Egg white	0.52 (0.18-1.5)	0.32 (0.16-0.55)	.47
Ovomucoid	0.46 (0.12-0.97)	0.20 (0.06-0.47)	.26

The Fisher exact test and Mann-Whitney *U* test were used.

*IQR*, Interquartile range.

### Clinical outcomes

In the OIT group, the proportions of participants who achieved STU to 3100 mg of egg protein were 20% at 1 year, 35% at 2 years, and 55% at 3 years. Within 3 years, 80% of the participants (16 of 20) achieved desensitization to 1000 mg of egg protein, whereas 5% (1 of 20) could not reach 1000 mg of egg protein on account of adverse reactions; 15% of the participants (3 of 20) dropped out: 2 participants withdrew from the study owing to persistent adverse reactions, and 1 participant was unable to continue receiving medical care because of relocation. In the historical control group, the proportions of patients who passed OFC to 3100 mg of egg protein were 0% at 1 year, 0% at 2 years, and 5% at 3 years, and the rates of passing OFC were significantly higher in the OIT group than in the historical control group at years 2 and 3 (at 2 years, *P* = .008; at 3 years, *P* = .001 [Table II]). Patients who passed OFC with 3100 mg of egg protein were permitted consume processed foods equivalent to half an egg at home.

**Table II:** Comparison of clinical outcomes of passing OFC to 3100 mg of egg protein

Time from baseline OFC, no. (%)	OIT group (n = 20)	Historical control group (n = 20)	*P* value
1 y	4 (20%)	0 (0%)	.11
2 y	7 (35%)	0 (0%)	.008
3 y	11 (55%)	1 (5%)	.001

The Fisher exact test was used.

### Adverse reactions

During hospitalization, 57 of the total 79 doses (72.2%) resulted in symptoms, and 40.5% of the participants (32 of 79) experienced adverse reactions requiring treatment (Table III). One participant required intramuscular adrenaline for severe abdominal pain and repetitive emesis during hospitalization. At home, the rate of symptoms per number of intakes was 15.9% (1,646 of 10,384), and moderate or severe symptoms occurred in 0.9% (97 of 10,384) and 0.04% (4 of 10,384) of participants, respectively. Anaphylaxis developed in 0.05% of ingestions (5 of 10,384); however, in 4 cases, symptoms disappeared immediately after treatment with an oral antihistamine, oral steroid, and inhalation of a β_2_ stimulant. One participant experienced severe abdominal pain and dyspnea but promptly improved after using an adrenaline autoinjector. All severe adverse reactions occurred in children with better adherence to the timing of consuming cooked egg powder every day, and they were not related to deviations from the protocol (see Table E3 in the Online Repository at www.jaci-global.org). None of the patients had symptoms suggestive of eosinophilic esophagitis. During the study period, 6 patients in the 2 groups developed symptoms resulting from accidental exposure to eggs. One patient in the historical control group and no patients in the OIT group developed anaphylaxis as a result of accidental exposure. The frequency of accidental exposure to eggs and associated anaphylaxis was not significantly different between the 2 groups.

**Table III:** Adverse reaction during the OIT protocol

Adverse reaction	In the hospital	At home
Total no. of intakes of OIT	79	10384
Adverse symptoms, no (%)	57 (72.2%)	1646 (15.9%)
Mild	27 (34.2%)	1509 (14.5%)
Moderate	28 (35.4%)	97 (0.9%)
Severe	1 (1.3%)	4 (0.04%)
Organ system of symptoms, no (%)		
Skin	16 (20.3%)	366 (3.5%)
Mucosal	28 (35.4%)	883 (8.5%)
Respiratory	10 (12.7%)	156 (1.5%)
Gastrointestinal	46 (58.2%)	502 (4.8%)
Cardiovascular	0 (0%)	0 (0%)
Neurologic	1 (1.3%)	20 (0.2%)
Anaphylaxis	7 (8.9%)	5 (0.05%)
Total no. of treatments, no (%)	32 (40.5%)	165 (1.6%)
Antihistamine	32 (40.5%)	157 (1.5%)
Corticosteroid	14 (17.7%)	31 (0.3%)
β_2_ inhalation	7 (8.8%)	37 (0.4%)
Adrenaline	1 (1.3%)	1 (0.01%)
Eosinophilic esophagitis, no (%)	0 (0%)	0 (0%)

### Immunologic changes

**Fig 3:**
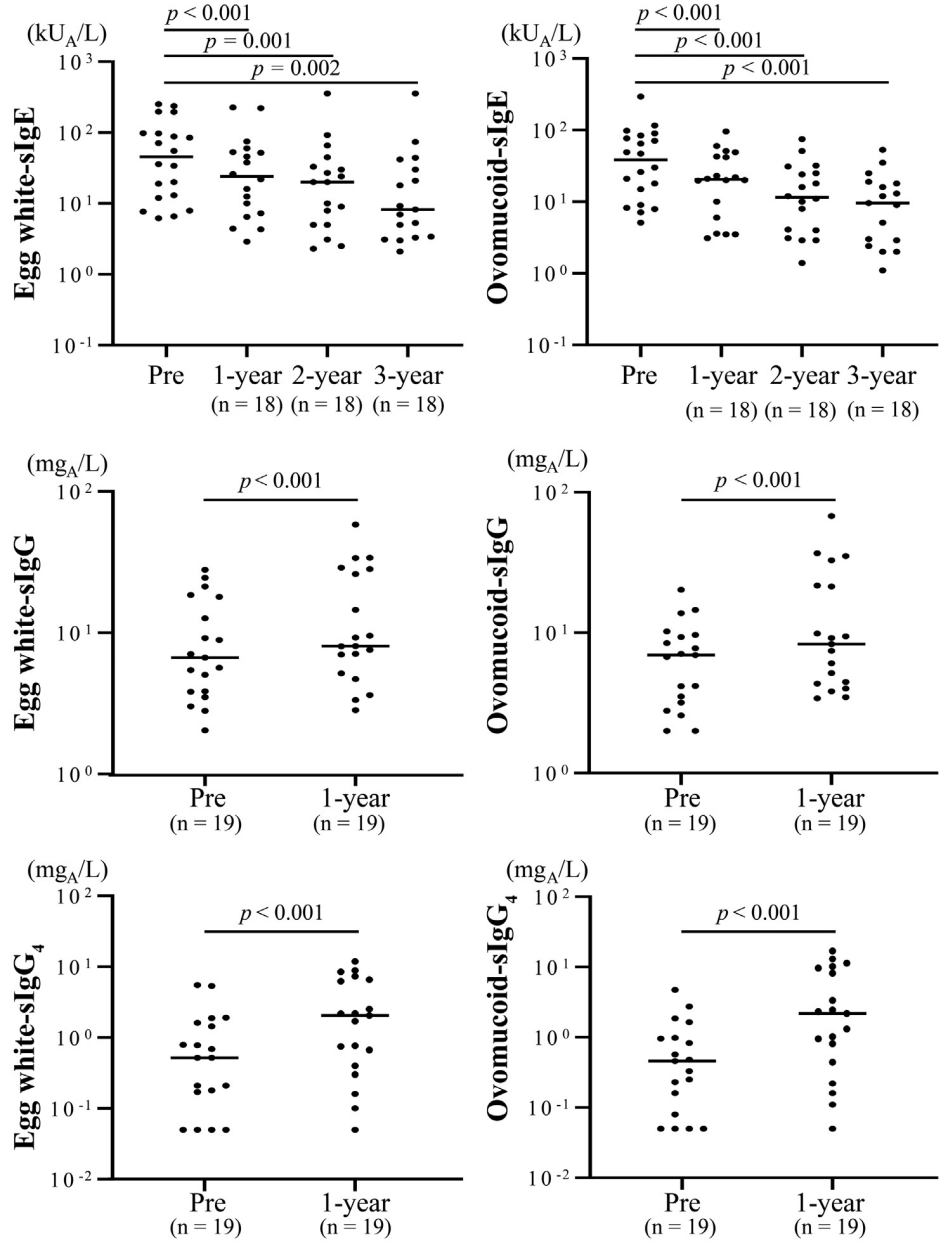
Changes in specific IgE, IgG, and IgG_4_ levels over time in the OIT group. Wilcoxon rank sum test. *Pre*, At baseline; *sIgE*, specific IgE; *sIgG*, specific IgG.

**Fig E2:**
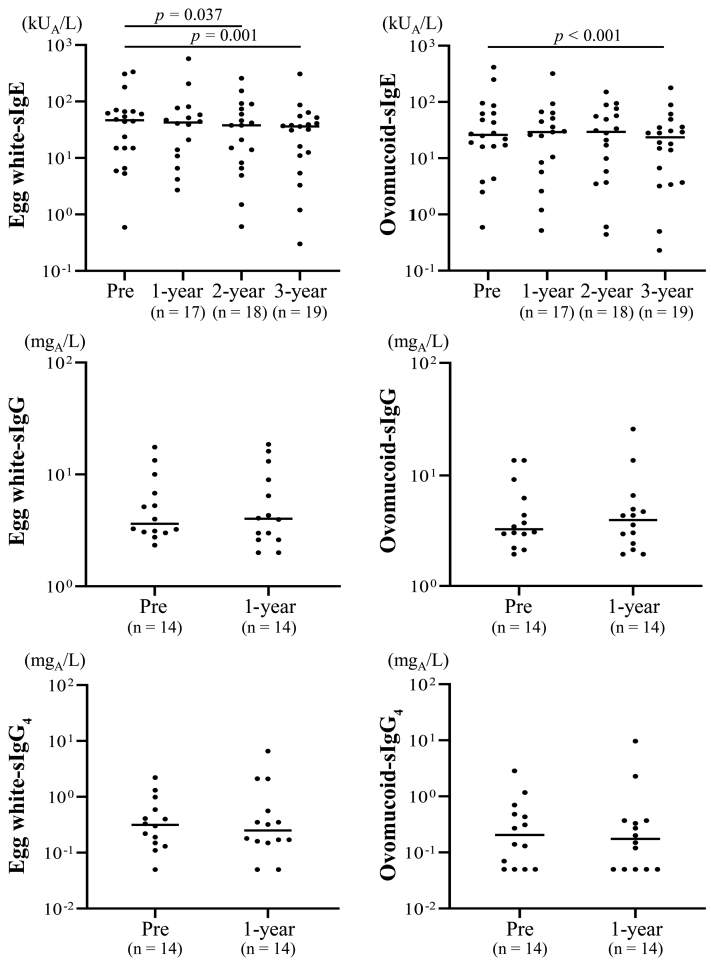


At 3 years, the median egg white- and ovomucoid-specific IgE levels in the OIT group were significantly lower than the levels at baseline (egg white, *P* = .002; ovomucoid, *P* < .001 [Fig] 3]). The median egg white- and ovomucoid-specific IgE levels in the historical control group decreased significantly after 3 years (egg white, *P* = .001; ovomucoid, *P* < .001 [see Fig E2 in the Online Repository at www.jaci-global.org]). Meanwhile, the rates of reduction in egg white- and ovomucoid-specific IgE levels from the start of OIT to 3 years were significantly higher in the OIT group than in the historical control group (egg white, *P* = .03; ovomucoid, *P* = .002 [see Table E4 in the Online Repository at www.jaci-global.org]). In contrast, the median egg white- and ovomucoid-specific IgG and IgG_4_ levels increased significantly from baseline to the 1-year mark in the OIT group (in the case of IgG: egg white, *P* < .001; ovomucoid, *P* < .001; in the case of IgG_4_: egg white, *P* < .001; ovomucoid, *P* < .001 [Fig 3]); however, the levels in the historical control group did not change (see Fig E2 in the Online Repository at www.jaci-global.org).

## Discussion

This is the first study to examine the long-term outcomes of OIT for children with anaphylactic egg allergy. Long-term OIT induced immunologic changes over time, increasing the rate of achievement of STU over time (to 55% after 3 years). After 3 years, the rate of passing OFC with 3100 mg of egg protein was significantly higher for the OIT group than for the historical control group (5%).

In 2 recent studies of long-term egg OIT, participants with severe anaphylaxis were excluded, and the median egg white-specific IgE levels were 10.3 and 15.6 kU_A_/L.^16^^,^^21^ In the current study, the median egg white-specific IgE level was 45.5 kU_A_/L; moreover, only children with a history of anaphylaxis were included; therefore, more participants with severe reactions were enrolled than reported in previous studies. Jones et al^16^ have reported on OIT for 4 years with a maintenance dose of 2000 mg of egg protein; they evaluated sustained unresponsiveness (SU) to 10 g of egg white and demonstrated efficacy over time. In the current study, the maintenance dose (1000 mg of egg protein) was set lower than in the previous study because the participants had developed anaphylaxis in response to egg. The numbers of participants who achieved STU gradually increased over time (4 of 20 at 1 year, 7 of 20 at 2 years, and 11 of 20 at 3 years), as in previous studies (11 of 40 at 2 years and 18 of 40 at 3 years).^16^ As a result, many participants were permitted to consume a variety of processed foods, equivalent to half an egg.

Regarding adverse reactions during OIT, in the current study with a maintenance dose of 1000 mg of egg protein, the rate of adverse reactions requiring treatment at home was 1.5%, and the rate of moderate or severe adverse reactions was 0.9%. In terms of treatment safety, our results were comparable to those of a previous study with a maintenance dose of 2000 mg of egg protein. In that study, 3.6% of the participants with adverse reactions required treatment for 2 years, 1.6% required treatment for 3 to 4 years, and the incidence of moderate symptoms was 0.7%.^15^^,^^16^ Although the current study included participants at higher risk than in previous studies,^16^^,^^21^ the frequencies of adverse reactions were comparable. This was achieved by reducing the maintenance dose to half that utilized in the previous study. In our previous study of even lower maintenance doses (194 mg) in children who had experienced symptoms of similar severity, severe adverse reactions were absent.^20^ However, the current study showed some severe adverse reactions; therefore, the maintenance dose of OIT for anaphylactic egg allergy may need to be lowered in consideration of risk. In addition, unlike in some other studies,^15^, ^16^, ^17^, ^18^, ^19^ in this study the dose was increased at home. Five patients developed anaphylaxis; however, it did not occur on the day on which the dose was increased at home. Premedicating with antihistamines, not increasing the dose during the first month after initiation, and increasing the dose slowly may explain why the dose could be increased safely. We have successfully implemented the same 3-year protocol as used in this study for milk and wheat OIT.^9^^,^^28^ As for eosinophilic esophagitis, most cases of eosinophilic esophagitis reported so far have been caused by egg OIT at high doses.^19^^,^^36^ A report on OIT for peanut allergy has suggested the possibility of dose dependence.^37^ Because the OIT in the current study was also performed at a relatively low maintenance dose and no participants developed eosinophilic gastrointestinal symptoms, the OIT for egg allergy may also be dose dependent. Regarding the number of accidental exposures, there was no difference between the 2 groups. In the previous peanut and wheat OIT reports,^9^^,^^38^ the numbers of allergic reactions due to accidental exposure were significantly higher in the control group than in the OIT group. Egg allergy is known to be more likely to acquire tolerance in the natural course than are those with other antigens, and in the current study, because 55% of those in the historical control group were able to consume a low-dose of egg after 3 years, symptoms may not have occurred after accidental exposure involving very a low dose of egg.

Concerning immunologic changes, the current study reported that the levels of egg white- and ovomucoid-specific IgE decreased whereas the levels of egg white- and ovomucoid-specific IgG and IgG_4_ increased during OIT; similar trends have been reported in previous studies.^15^^,^^16^^,^^18^, ^19^, ^20^ Interestingly, both the OIT and historical control groups showed a significant decrease in egg white- and ovomucoid-specific IgE levels after 3 years as compared with baseline, whereas the rates of reduction were significantly higher in the OIT group. Thus, the OIT group has accelerated immunologic changes compared with those in the historical control group.

As for the form of egg used, all of the previous long-term follow-ups of OIT have used powdered eggs.^16^^,^^21^ The current study also used cooked egg powder, and no participants withdrew on account of refusal to consume the powder. Children with anaphylactic egg allergy require long-term treatment, and long-term OIT requires high adherence. Therefore, the use of powder reduces the burden on patients’ families.

The first limitation of this study is that it was not a randomized controlled study. Randomization was not feasible because the children visited our hospital to receive OIT.^39^ Therefore, we established a historical control group. Although age was significantly lower in the historical control group, egg allergy tends to be resolved at a younger age.^5^^,^^7^ Nevertheless, the rate of passing the 3100-mg OFC in the OIT group was significantly higher than that in the control group. Therefore, the age difference did not affect the results. Furthermore, the historical control group did not completely eliminate egg intake for ethical reasons, and participants were permitted to consume the amount confirmed to be negative OFC result.^31^ As a result, the historical control group was able to consume very few eggs over the 3 years, and the effect on the results was considered minimal. In addition, although the detailed role of specific IgG in immediate food allergy is not yet known,^40^ we cannot exclude the possibility that the nonrandomization caused a trend toward higher specific IgG levels in the OIT group, which may have affected the results.

The second limitation was the exclusion of study participants with no history of anaphylaxis. Two participants who underwent OIT and 7 patients who did not undergo OIT had no history of anaphylaxis; however, the results of achievement of STU and immunologic changes were similar even if these participants were included.

The third limitation was that we assessed STU on the basis of OFC with 2 weeks of avoidance. Other OIT trials^8^, ^9^, ^10^^,^^12^, ^13^, ^14^^,^^20^^,^^28^ have used a 2-week avoidance to assess STU, whereas SU is usually determined by OFC after avoidance for more than 1 month.^21^ However, participants who are able to consume 3100 mg of egg protein after a 2-week avoidance were able to safely resume intake after short interruptions owing to situations such as a common cold. This means that they will be able to safely consume a variety of processed foods containing 3100 mg of egg protein in their daily life, which would improve their quality of life.^4^^,^^35^ In fact, the children who passed the 3100-mg OFC were permitted to consume various processed foods.

The final limitation was the use of 3 different egg products (powder, cake, and hamburger) with an OFC of 3100 mg of egg protein. In the current study, 17 participants (85%) used cooked egg powder, except for 1 for cake and 2 for hamburgers. Two participants who passed OFC with 3100 mg of egg protein other than the powder were also able to consume processed products equivalent to 3100 mg of egg protein at home without adverse reactions; thus, the influence of the 3 products on the results is considered to be minimal.

In conclusion, long-term OIT induced immunologic changes over time in almost all children and led to an STU of 3100 mg of egg protein in half of those with anaphylactic egg allergy within 3 years. Moreover, the current study was performed with safety comparable to that of the previous study by setting the maintenance dose at a lower dose. However, severe adverse reactions were observed during the induction and maintenance phases; thus, future studies with a further reduced maintenance dose or slowly increased doses are warranted to identify a safer protocol.

**Clinical implications**

Long-term oral immunotherapy for egg allergy with anaphylaxis is shown to accelerate immunologic changes over time and increase the amount of egg that can be ingested.

## Supplementary data

Table E1-E4
