# Supplementary material for: Long-term outcomes of oral immunotherapy for anaphylactic egg allergy in children
Source: J Allergy Clin Immunol Glob. 2022 Apr 30;1(3):138–44. doi: 10.1016/j.jacig.2022.03.005 (PMC10509875; doi:10.1016/j.jacig.2022.03.005)
Supplement: Table E1-E4 [file mmc1.docx]

**TABLE E1.** **Classification of severity according to clinical symptoms**

|  | 1 (mild) | 2 (moderate) | 3 (severe) |
| --- | --- | --- | --- |
| Skin | Localized urticaria, exanthema, wheal, pruritus | Generalized urticaria, exanthema, wheal, pruritus | - |
|  | Swollen eyelid or lip | Swollen whole face | - |
| Gastrointestinal tract | Pruritus of the throat or oral cavity | Throat pain | - |
|  | Mild abdominal pain | Moderate abdominal pain | Cramps |
|  | Nausea, emesis, diarrhea | Recurrent emesis, diarrhea | Continuous emesis, loss of bowel control |
| Respiratory tract | Intermittent cough, nasal congestion, sneezing, rhinorrhea | Repetitive cough | Persistent cough, hoarseness, "barking" cough |
|  | - | Chest tightness, mild wheezing | Apparent wheezing, dyspnea, cyanosis, saturation < 92%, swallowing or speaking difficulties, throat tightness,  respiratory arrest |
| Cardiovascular | - | Pale face, mild hypotension^*^, tachycardia (increase > 15 beats/min) | Hypotension^**^, dysrhythmia, severe bradycardia, cardiac arrest |
| Neurological | Change in activity level, tiredness | "Light-headedness," feeling of "pending doom," somnolence | Confusion, loss of consciousness, incontinence |

*Mild hypotension: < 80+ (2×age)] mmHg in age of 1-10

**Hypotension: < 70+ (2×age)] mmHg in age of 1-10

This severity score was defined according to the anaphylaxis guidelines for Japan.^1,2,3^

**TABLE E2. Dosing schedule for egg protein in oral immunotherapy**

| STEP | 1 | 2 | 3 | 4 | 5 | 6 | 7 | 8 |
| --- | --- | --- | --- | --- | --- | --- | --- | --- |
| Egg protein (mg) | 62.5 | 125 | 187.5 | 250 | 375 | 500 | 750 | 1000 |
| Total amount of powder (mg) | 250 | 500 | 750 | 1000 | 1500 | 2000 | 3000 | 4000 |

**TABLE E3. Detail of severe adverse reaction at home**

| Case | Age (year) | Sex | Baseline  egg white  -sIgE (kU_A_/L) | Time of symptom onset (month) | Symptom | Treatment | Outcome |
| --- | --- | --- | --- | --- | --- | --- | --- |
| 1 | 6 | Male | 252 | 1 | Severe abdominal pain | Oral antihistamines and steroids | 3100 mg STU |
| 2 | 6 | Female | 13.2 | 16 | Severe abdominal pain | Oral antihistamines and steroids | Desensitization |
| 3 | 6 | Female | 238 | 14 | Apparent wheezing | β_2_-stimulant inhalation | Desensitization |
| 4 | 8 | Male | 20.3 | 1 | Severe abdominal pain and dyspnea | Adrenalin autoinjection,  β_2_-stimulant inhalation,  oral antihistamines and steroids | Drop out |

sIgE, specific immunoglobulin E; STU, short-term unresponsiveness.

In cases 1 and 4, the symptoms occurred during the induction period, without the day of dose escalation. In cases 2 and 3, symptoms appeared during the maintenance period, 3 and 6 months after desensitization, respectively.

**TABLE E4.** **Reduction rates in specific IgE over time in the OIT and historical control group**

| Specific IgE | OIT group  (n = 20) | Historical control group  (n = 20) | *p* value |
| --- | --- | --- | --- |
| Egg white | 62% (48-77) | 41% (21-54) | 0.03 |
| Ovomucoid | 72% (62-80) | 35% (23-59) | 0.002 |

IgE, immunoglobulin E; OIT, oral immunotherapy. Mann–Whitney U test, Median (IQR). The reduction rate over time was calculated by dividing the specific IgE level after 3 years by the baseline.

**References: Table E-1**

1. Yanagida N, Okada Y, Sato S, Ebisawa M. New approach for food allergy management using low-dose oral food challenges and low-dose oral immunotherapies. Allergol Int 2016;65:135-40.
2. Ebisawa M, Ito K, Fujisawa T; Committee for Japanese Pediatric Guideline for Food Allergy, The Japanese Society of Pediatric Allergy and Clinical Immunology, The Japanese Society of Allergology. Japanese guidelines for food allergy 2017. Allergol Int 2017;66:248-64.
3. Yanagida N, Minoura T, Kitaoka S, Ebisawa M. A three-level stepwise oral food challenge for egg, milk, and wheat allergy. J Allergy Clin Immunol Pract 2018;6:658-60.e10.
