# Supplementary material for: Long-term outcomes of oral immunotherapy for anaphylactic egg allergy in children
Source: J Allergy Clin Immunol Glob. 2022 Apr 30;1(3):138–44. doi: 10.1016/j.jacig.2022.03.005 (PMC10509875; doi:10.1016/j.jacig.2022.03.005)
Supplement: Figure legends [file mmc2.docx]

FIGURE E1. Historical control group

Participants underwent stepwise OFC with 250 mg, 1000 mg, and 3100 mg of egg protein according to the Japanese Food Allergy Guidelines to confirm the egg threshold within 3 years.

OFC, oral food challenge

FIGURE E2. Changes in specific IgE, IgG, and IgG_4_ over time in the historical control group

sIgE, specific immunoglobulin E; sIgG, specific immunoglobulin G; sIgG_4_, specific immunoglobulin G_4_

Wilcoxon’s rank-sum test. Pre = at baseline.

**Graphical abstract legend**

This study investigates the long-term outcomes of OIT for anaphylactic egg allergy. Long-term egg OIT increased the rate of achieving STU over time: 1 year, 20%; 2 years, 35%; 3 years, 55%. After 3 years, the rate of passing the OFC with 3100 mg of egg protein was significantly higher for the OIT group than for the historical control group (5%).

Abbreviation: OFC, oral food challenge; OIT, oral immunotherapy; sIgE, specific immunoglobulin E; sIgG, specific immunoglobulin G; sIgG_4_, specific immunoglobulin G_4_; STU, short-term unresponsiveness
